# Institutional support for breastfeeding in Ghana: a case study of University of Education, Winneba

**DOI:** 10.1186/s13104-018-3608-y

**Published:** 2018-07-24

**Authors:** Jacqueline Nkrumah, Fred Yao Gbagbo

**Affiliations:** Department of Health Administration and Education, Faculty of Science Education, University of Education, Winneba, P.O Box 25, Winneba, Central Region Ghana

**Keywords:** Institutional support, Breastfeeding, Childcare, Academic work, Student-mothers

## Abstract

**Objectives:**

This study explored institutional support for breastfeeding student-mothers in the University of Education, Winneba, Ghana. It also examined challenges associated with combining academic work with breastfeeding and childcare.

**Results:**

Findings show that although the University as an institution does not have any formal system in place to support breastfeeding among student-mothers, it does follow the provisions made for breastfeeding under the maternity protection section of the labor Act (Act, 651) for its employees. Consequently, breastfeeding student mothers use under trees, lobbies, and Junior Common Rooms of on-campus halls of residence as lactation sites which exposes their babies to risk of infection. The absence of support put student-mothers through stress, divided attention, and conflicting responsibilities between academic work and childcare. Further studies to investigate the situation on other university campuses are recommended to promote policy and interventions on breastfeeding and childcare in tertiary institutions in Ghana to enable students maintain a balance between breastfeeding, childcare and academic work.

**Electronic supplementary material:**

The online version of this article (10.1186/s13104-018-3608-y) contains supplementary material, which is available to authorized users.

## Introduction

The general decline in breastfeeding worldwide in the early 1970s drew attention of international organizations such the World Health Organization (WHO) and the United Nations Children Fund (UNICEF) to formulate policies that would help promote and maintain breastfeeding practice [1–4]. Consequently, various policy recommendations and declarations have been formulated to promote and protect breastfeeding. The International Labor Organization (ILO) has also emphasized the importance of breastfeeding in its maternity protection conventions [2, 3, 5–7].

Global studies on breastfeeding and employment reveals that women who plan to return to work after delivery may either not breastfeed or wean their children earlier than expected as compared to their counterparts who are not employed or work on part-time basis [8–11]. Studies in countries with legislation on workplace support for breastfeeding have found that absence of organizational policies, breastfeeding facilities and information on organizations’ support for breastfeeding negatively impact on the practice [12–14].

In Ghana, apart from the maternity protection provision of the labor Act (Act 651 of 2003) there are no explicit public policies on workplace support for breastfeeding [15]. In the educational sector, available records indicate that the number of females enrolling into tertiary programs increased by 8.3% between 2005 and 2014 [16]. However, Policies on tertiary institutions’ support for breastfeeding are nonexistent. Anecdotal evidence shows that students in tertiary institutions in Ghana have reproductive intentions, hence a significant number of them are either mothers or likely to become mothers in the course of their study posing challenges to breastfeeding, childcare and schooling just like their counterparts in formal employment. This study sought to examine challenges encountered combining breastfeeding and childcare with academic work to inform policy decisions on breastfeeding support.

## Main text

### Methods

The study design was qualitative case study using focus group discussions and non-directive interviews.

#### Study institution

University of Education, Winneba was established in 1992 to train middle and top-level manpower for the educational sector of Ghana. The University has four main satellite campuses, (Winneba and Ajumako in the Central Region of Ghana, Kumasi, and Mampong campuses in Ashanti Region of Ghana). The Winneba campus has three smaller campuses with a total student population of 18, 987 as at 2016/2017 academic year. Female students constituted 30% of the total student population. The Vice-Chancellor, Registrar and other Principal Officers operate from the Winneba Campus while the other three campuses are managed by Principals under the supervision of the Vice-Chancellor.

#### Study population and sampling

The study population included all women in their reproductive age resident in Effutu Municipality and all unit heads in University of Education, Winneba at the time of the study. A sample of 30 breastfeeding student-mothers between the ages of 18–49 years and 3 unit heads (human resource, gender main streaming, and planning) were respectively selected for the study through snowballing and purposive sampling. Four participants identified at a child welfare clinic on the University Campus were initially recruited to support the authors to recruit 32 more participants of which 30 consented to partake in the study. To participate in the study, a respondent must be a breastfeeding student mother or Unit Head.

#### Data collection

The study was conducted between January and May, 2017. Focus group discussions and non-directive interviews were used for data collection. Participants were divided into six groups of five members in each group and a venue was agreed for the discussions. The study objectives and rules of discussions were communicated to each participant and a written consent obtained from each of them prior to each session. Appointments were also booked with the various unit heads for the face-to-face interviews.

The focus group discussions and interviews were done using interview and discussion guides developed by authors. These were based on guidelines from module 10 of the ILO’s requirements for breastfeeding arrangements at work and other relevant literature [7, 12, 14, 16]. (Please, refer to Additional file 1 for research instruments.) Both the interview and group discussion guides were pretested among four student-mothers and two administrators in the university. Research instruments were also shared with expertise in this area for content validity. The focus group discussions were moderated by first author. Participants were randomly assigned to focus groups and the identity of moderator was concealed until the time for the discussions. Data was collected through field notes and audio recording. Data collection and recordings were both in English language.

#### Data analysis

Data were analyzed using content analysis and descriptive statistics between April and May, 2017. Data from the audio recorder was transcribed by first author. The field notes and transcribed audio recordings were converted into typed script in a two column table format (the first column contained the script and the second column for making notes). The script was read through three times by second author and brief notes on revealing information were made in the second column. Both authors thoroughly read through the notes to identify and document relevant information which were grouped into broad themes. These themes were checked, discussed, and revised twice by authors before arranging them into categories and subcategories. The main categories and subcategories were further compared to the typed script to ensure that none of the relevant information was missing. Descriptive analysis was done using Microsoft Excel (windows 10.0).

### Results

Table 1 presents the background characteristics of respondents in the study. The mean age of mothers and babies are 31 ± 6 and 5 ± 5 respectively. Eighty percent of the respondents were married and about 66% were undergraduate students.

**Table 1:** Background characteristics of participants, N = 30

Characteristics	Frequency	%
Age of mother (years)		
23–27	6	20.0
28–32	12	40.0
33–37	8	26.7
38–42	4	13.3
Total	30	100
Mean age 31 ± 6		
Age of baby (months)		
0–3	8	26.7
4–7	11	36.7
8–11	6	20.0
12+	5	16.6
Total	30	100
Mean age of baby 5 ± 5		
Marital status		
Married	24	80.0
Single	6	20.0
Divorce	0	0.0
Widow	0	0.0
Total	30	100
Parity		
1	4	13.3
2	8	26.7
3	12	40.0
4	6	20.0
Total	30	100
Religion		
Christian	21	70
Muslim	9	30
Others	0	0.00
Total	30	100
Program		
Diploma	6	20.0
Undergraduate	20	66.7
Graduate	4	13.3
Total	30	100
Ethnicity		
Akan	12	40.0
Ewe	4	13.3
Ga	3	10.0
Others	11	36.7
Total	30	100

Constructed by authors using field data; analyzed using Microsoft Excel

#### University’s support for breastfeeding

Interviews with unit heads showed that the university complies only with provisions on maternity protection in the Labor Act (Act 651 of 2003). Key issues of concern to student-mothers are in Table 2.

**Table 2:** Support for breastfeeding student-mothers, N = 30

Main category	Subcategories
University support for breastfeeding	Breastfeeding policy, lactation rooms/onsite crèches Environmental hazards, mothers’ preferences
Reasons for combing academic work and childcare	Ageing, fear of losing admission Pressure to meet social expectation
Challenges of combining childcare with academic work	Conflicting responsibility, compromised child safety, divided attention, stress
Mechanisms for coping with childcare and academic work	Effective time management Support from peers, academic counselors, relatives and spouses. Use of crèches

Constructed by authors using field data. Analyzed using content analysis

Mothers lamented about cost of keeping nannies and expressed preferences for onsite crèche which is more cost effective and safer than use of nannies. A participant indicated the following:

*…never have I sighted anything called lactation room. I always breastfeed under trees during lecture times. ……sending my first baby to crèche boosted my baby’s motor skills.* (*Post graduate; Student*- *mother*). Please, see additional illustrative quotes from student-mothers in (Additional file 2: Appendix S1).

#### Reasons for combining academic work and childcare

Ageing, fear of losing admission and pressure from in-laws were reasons provided for combining academic work and child care. A participant indicated that:

*….after a year in marriage, my in*-*laws expected a baby, put pressure on me and tag me as barren………*(*Level 200; Student* -*mother*). Please, see additional illustrative quotes from student-mothers in (Additional file 2: Appendix S1).

#### Challenges of combining childcare and academic work

This category comprises divided attention, conflicting responsibility and stress. Participants indicated that demands from babies and academic activities are stressful. A respondent indicated that:

*…..when my nanny calls and reports of my baby’s cry, I suddenly lose focus in class and leave whiles lectures are going on to care for my baby……. (Level 300; Student*- *mother).* Please, see additional illustrative quotes from student-mothers in (Additional file 2: Appendix S1).

#### Mechanisms for coping with family life and academic work

Participants enumerated time management, peers/relatives support, academic counseling and use of crèches as key coping mechanisms for childcare and academic work. A respondent said:

*The motivation I received from my academic counselor really encouraged me to adopt all possible means of combining mothering and learning to excel in my academic work. This changed my mind from deferring the course due to child birth. (Post graduate Diploma; Student* -*Mother)* Please, see additional illustrative quotes from student-mothers in (Additional file 2: Appendix S1).

#### Effect of support on breastfeeding

Respondents who have had at least two children prior to admission mentioned that exclusive breastfeeding is cost effective and improved their babies’ health. Almost all mothers expressed the desire for exclusive breastfeeding amidst the challenges. A respondent indicated:

*…so when will the University provide a decent place for breastfeeding babies instead of paying more attention to spiritual places of worship on campus…? (Level 400; Student*- *mother).* Please, see additional illustrative quotes from student-mothers in (Additional file 2: Appendix S1).

### Discussion

The quest for higher educational pursuit and/or personal development was observed as one of the reasons for combining child care and schooling. Because children are perceived as blessing in Ghanaian societies, women are pressured to have children at a certain age. This observation is consistent with previous findings that a woman’s age has implications for pregnancy [17–19].

Our study observed that, a driving force compelling students to have children whiles in school was the perceived limited time available to achieve their reproductive and academic intentions. This supports a study from northern Ghana where childless women suffer discrimination, stigma and ostracism making life without children not worth living [20]. Fatigue from childcare and academic work has also been reported in similar studies as contributing to less life satisfaction [21–24].

There are various legislations on institutional support for breastfeeding in many countries [25–27]. The provision of paid maternity leave, flexible work schedule, breastfeeding breaks, and ‘half-day’ are examples of workplace support for breastfeeding employee-mothers in Ghana. However, this provision excludes students since they are not employees. The absence of breastfeeding support for student-mothers in tertiary institution could also be partly due to lack of national policies to support breastfeeding among student-mothers. Thus, a student mother who delivers a baby in the course of the academic year could only defer the course to a later time if she so desire but not supported on campus to encourage exclusive breastfeeding in a suitable environment. This finding is consistent with others from England which reported use of unsuitable places such as toilet cubicles and shower rooms as lactation rooms [28].

There were no vast differences in the responses obtained from the various levels of student-mothers. The challenges of combining childcare with academic work were mostly work overload resulting in emotional and physical stress on these respondents. Some of the respondents believed that the situation would have been different if heads and directors in the university have ever had such experiences. Whiles undergraduate student-mothers resorted to physical and emotional support from peers and significant others, those at the post graduate levels relied on in-laws, husbands and traders on campus to support their childcare in rented apartment although quite expensive. Findings of this study confirm others that institutional supports are essential to continuous breastfeeding [29, 30]. Where feasible, priority should be given to breastfeeding student mothers and pregnant students during allocation of hostel facilities on campus to facilitate childcare and academic work. Further studies will be required to examine the situation on other university campuses to inform national policy and programs on breastfeeding and childcare in tertiary institutions in Ghana.

## Limitations of the study

The study has the following limitations:

1. This study involved only regular students of the Winneba campus hence findings cannot be generalized to all public universities in Ghana.
2. The total number of breastfeeding student mothers identified during the study was 30. This may have some implications for policy decisions.

## Additional files

**Additional file 1.** The data collection instrument consists of interview and focus group discussions guides used in collecting data for the study.

**Additional file 2: Appendix S1.** The appendix I contains further illustrative quotes from participants of the study.

## Abbreviations

GDHS: Ghana Demographic and Health Survey
ILO: International Labor Organization
UEW: University of Education, Winneba
UN: United Nations
UNICEF: United Nations Children’s Fund
WHO: World Health Organization

## References

[1] Eckhardt KW, Hendershot GE (1984). Analysis of the reversal in breast feeding trends in the early 1970s. Public Health Rep.

[2] World Health Organization. Global strategy for infant and young child feeding. Geneva: World Health Organization. 2003. http://apps.who.int/iris/bitstream/10665/42590/1/9241562218.pdf. Accessed 29 June 2017.

[3] UNICEF. Celebrating the Innocenti declaration on the protection, promotion and support of breastfeeding. Past achievements, present challenges and the way forward for infant and young child feeding. Florence: UNICEF Innocenti Research Centre. 2005. http://www.unicef.org/nutrition/files/Innocenti_plus15_BreastfeedingReport.pdf. Accessed 13 June 2017.

[4] World Health Organization. Joint WHO/UNICEF meeting on infant and young child feeding: statement recommendations list of participants. Geneva: World Health Organization. 1979. http://apps.who.int/iris/bitstream/10665/62980/1/1580_1979_eng.pdf. Accessed 29 June 2017.

[5] World Health Organization (1981). International code of marketing of breast-milk substitutes.

[6] World Health Organization/United Nations Children Fund. Baby-Friendly Hospital Initiative. Revised, updated and expanded for integrated care. 2009. http://www.who.int/nutrition/publications/infantfeeding/bfhi_trainingcourse/en/. Accessed 29 June 2017. 23926623

[7] International Labor Organization. Maternity protection resource package—from aspiration to reality for all. Module 10: breastfeeding arrangements at work. International Labor Organization. 2012. http://mprp.itcilo.org/allegati/en/m10.pdf. Accessed 1 Dec 2016.

[8] Scrimshaw S, Engle P, Arnold L, Haynes K (1987). Factors affecting breastfeeding among women of Mexican origin or descent in Los Angeles. Am J Public Health.

[9] Kurinu K, Shiono PH, Ezrine SF, Rhaods GG (1989). Does maternal employment affect breast-feeding?. Am J Public Health.

[10] Lindberg LD (1996). Women’s decisions about breast-feeding and maternal employment. J Marriage Fam.

[11] Fein SB, Roe B (1998). The effect of work status on initiation and duration of breast-feeding. Am J Public Health.

[12] Brown AC, Poag S, Kasprzycki C (2001). Exploring large employers’ and small employers’ knowledge, attitudes, and practices on breastfeeding support in the workplace. J Hum Lact.

[13] Weber D, Janson J, Michelle Nolan M, Ming Wen L, Rissel C (2011). Female employees’ perceptions of organizational support for breastfeeding at work: findings from an Australian health service workplace. Int Breastfeed J.

[14] Bar-Yam NB (1998). Workplace lactation support, part I: a return-to-work breastfeeding assessment tool. J Hum Lact.

[15] Labour Act. (Act 651). Republic of Ghana. 2003.

[16] Index Mundi. Ghana—School enrollment. Undated. https://www.indexmundi.com/facts/ghana/school-enrollment. Accessed 28 June 2017.

[17] Cleary-Goldman J, Malone FD, Vidaver J, Ball RH, Nyberg DA, Comstock CH, Saade GR, Eddleman KA (2005). Impact of maternal age on obstetric outcome. Obstet Gynecol.

[18] Jacobsson B, Ladfors L, Milsom I (2004). Advanced maternal age and adverse perinatal outcome. Obstet Gynecol.

[19] Bayrampour M, Heaman M, Duncan KA, Tough S (2012). Advanced maternal age and risk perception: a qualitative study. BMC Pregnancy Childbirth.

[20] Cui W (2010). Mother or nothing: the agony of infertility. Bull World Heath Organiz..

[21] Tabong FT, Adongo FB (2013). Understanding the social meaning of infertility and childbearing: a qualitative study of the perception of childbearing and childlessness in northern Ghana. PLoS ONE.

[22] Addofo, S. Challenges and coping strategies of student nursing mothers in tertiary institutions in the greater Accra region of Ghana. 2013. http://ugspace.ug.edu.gh/bitstream/123456789/5428/1/Stella%20Adofo_Challenges%20and%20Coping%20Strategies%20of%20Student%20Nursing%20Mothers%20in%20Tertiary%20Institutions%20in%20the%20Greater%20Accra%20Region%20of%20Ghana_2013.pdf. Accessed 28 June 2017.

[23] Killien MG (2005). The role of social support in facilitating postpartum women’s return to employment. J Obstet Gynecol Neonatal Nurs.

[24] Grice MM, McGovern PM, Alexander BH (2008). Flexible work arrangements and work–family conflict after childbirth. Occup Med.

[25] McMeans, A. Government urged to double work breaks for breastfeeding mothers. 2009. https://www.thenational.ae/uae/government/government-urged-to-double-work-breaks-for-breastfeeding-mothers-1.487243. Accessed 25 May 2018.

[26] Drago R, Hayes J, Yi Y (2010). Better health for mothers and children: breastfeeding accommodations under the Affordable Care Act.

[27] Lactancia de madres trabajadoras es promovida por empresas publicas. 2008. http://radio.rpp.com.pe/saludenrpp/lactancia-de-madres-trabajadoras-es-promovida-por-empresas-publicas/. Accessed 12 Apr 2016.

[28] Sousan VS, Hosseinzadeh M, Mohammadi E, Hassankhani H, Fooladi MM (2016). Perceived stress in breastfeeding working mothers in Iran. Int J Med Res Health Sci.

[29] Kosmala-Anderson J, Wallace LM (2006). Breastfeeding works: the role of employers in supporting women who wish to breastfeed and work in four organizations in England. J Public Health.

[30] Esia-Donkoh K (2014). Child-rearing practices among student-mothers at University of Cape Coast, Ghana. Soc Biol Human Affairs.

