# Supplementary material for: Institutional support for breastfeeding in Ghana: a case study of University of Education, Winneba
Source: BMC Res Notes. 2018 Jul 24;11:501. doi: 10.1186/s13104-018-3608-y (PMC6057014; doi:10.1186/s13104-018-3608-y)
Supplement: Supplementary file 1 — Additional file 1. The data collection instrument consists of interview and focus group discussions guides used in collecting data for the study. [file 13104_2018_3608_MOESM1_ESM.docx]

**Research Instruments**

**Interview Guide for Data Collection on Institutional Support for Breastfeeding**

This guide is to assist researchers obtain information on Public universities support for breastfeeding and child care among employees and student mothers. We wish to state that data from this interview would be used for publication and education purposes. We would be grateful if your office could grant us interview to discuss the below mentioned questions.

**Interview Questions**

**(A)Breastfeeding Policy at Workplace**

1. Does the university have specific policies that support breastfeeding?
2. If yes, what are theprovisions outlined in the policy to enable:
3. employee-mothers to breastfeed their babies as prescribed by the WHO/Ghana Health Service protocols?
4. student-mothers to breastfeed their babies as prescribed by the WHO/Ghana Health Service protocols
5. If no, are there specific actions/practices that support breastfeeding in the university?

**(B) Breastfeeding Facilities**

1. Do you know or ever heard about lactation site/room before?
2. If yes, does the university have lactation rooms/sites for employees and student-mothers?
3. If the university has lactation sites/rooms, do you communicate this to students and staff?
4. How do you communicate this information?

**(C) Breastfeeding Support**

1. Does the university have lactation programs (i.e., flexible scheduling for milk expression, free supply of milk expression packs and educational programs such as breastfeeding, childbirth, and parenting seminars) that support and encourage employees and student- mothers to breastfeed?
2. Do the university’s general personnelresource materials, safety manuals or new employee orientation manuals, and students’ handbook contain information on any of the following?
3. leave entitlements
4. deferment of course due to child birth
5. announcements concerning potential flexible work options
6. childcare information and options
7. specific arrangements and facilities to support breastfeeding on return to work
8. specific arrangements and facilities to support breastfeeding among student-mothers

**(D) Breastfeeding Break**

1. Are breastfeeding employees allowed to observe breastfeeding breaks?
2. How are breastfeeding breaks scheduled for nursing mothers?
3. Are breastfeeding breaks considered as working hours and paid for accordingly?
4. Do breastfeeding employees gets sanctioned or loses income because of nursing breaks?

**(E) Maternity Leave**

1. Does the university grant maternity leave to expectant mothers?
2. What is the duration for maternity leave?
3. How does the university handle students who deliver in the course of their work?
4. Are students officially allowed to be in the university hostels with their new born babies?
5. If yes, are there special hostels with facilities that facilitate childcare on campus?
6. If no, has the university considered establishing mothers’ hostels on campus?

**GUIDE ON FOCUS GROUP DISCUSSION**

**Perception about exclusive breastfeeding**

1. Do you/did you practice exclusive breastfeeding?
2. If yes, did you/would you continued breastfeeding after six months?
3. Can you tell me why you practice/practicedexclusive/continuous breastfeeding?
4. What is your perception about exclusive breastfeeding?

**Reasons for combining childcare with academic work**

1. How long have you been in this university?
2. How many children have you since you joined this university?
3. How old is your current baby?
4. Have you heard about family planning?
5. Why did you choose to combine academic work with childcare at this time?

**Breastfeeding support to student mothers**

1. Do you reside on campus?
2. If yes, where exactly, and if no, why not?
3. Do you get any form of support from the university to help you breastfeed?
4. If yes, what are they?
5. Have you ever heard of lactation sites/rooms andonsite crèche on campus?
6. If yes, where are they located
7. If no are there improvise rooms designated for breastfeeding?
8. In what ways do you think availability of lactation sites/onsite crèche can facilitate breastfeeding and childcare on campus?

**Challenges in childcare and academic work**

1. Are there any challenges with breastfeeding, childcare and academic work?
2. In what ways do the following activities conflict with childcare and breastfeeding?
3. Lectures
4. Group studies/Discussions
5. Quizzes/ Examinations
6. Meeting deadlines for submitting assignments/term papers
7. What challenges do the absence of lactation sites/onsite crèches presents to breastfeeding and childcare on campus?

**Mechanisms for coping with childcare and academic work**

1. How are you able to combine breastfeeding and studies?
2. How are you able to cope with academic work and childcare?
3. How are you able to cope with the conflict between the following activities and childcare as well as breastfeeding on campus?
4. Lectures
5. Group studies/Discussions
6. Quizzes/ Examinations
7. Meeting deadlines for submitting assignments/term papers
8. How do you balance academic work with breastfeeding?
9. Do you have any further comments?
