# Supplementary material for: Institutional support for breastfeeding in Ghana: a case study of University of Education, Winneba
Source: BMC Res Notes. 2018 Jul 24;11:501. doi: 10.1186/s13104-018-3608-y (PMC6057014; doi:10.1186/s13104-018-3608-y)
Supplement: Supplementary file 2 — Additional file 2: Appendix S1. The appendix I contains further illustrative quotes from participants of the study. [file 13104_2018_3608_MOESM2_ESM.docx]

***Appendix: I***

**Additional Illustrative Quotes**

These have are grouped under the main themes.

***University support for breastfeeding***

One of the participants had this to say

*I always sit under shades or look for a summer hut to breastfeed my baby during lecture times.*

Another participant lamented that:

*..Exposing my breast just to feed my baby in the open has been a routine. As a moslem lady, can you imagine me covering my entire body as prescribed by my religion but expose my breast to ‘public viewing’ because there is no secluded breastfeeding room to ensure such privacy?... (Student mother)*

Another participant lamented that:

*..Exposing my breast just to feed my baby in the open has been a routine. As a Moslem lady, can you imagine me covering my entire body as prescribed by my religion but expose my breast to ‘public viewing’ because there is no secluded breastfeeding room to ensure such privacy?... (Level 400 Student -mother)*

***Reasons for combining academic work, breastfeeding and family childcare***

*…I’m getting older each day, hence had to combine childbirth and academic work in order to meet my reproductive and academic intentions…( Post graduate; Student- mother)*

***Challenges of combining breastfeeding with academic work***

Another reported that

*…. My mind simply becomes shut to academic work anytime I hear my baby crying outside whilst in class. Although I pretend most at times to be concentrating in class, its simply not possible but I just have to fake the concentration just to please my lecturer—(Student mother)*

Another reported that:

*…. My mind simply becomes shut to academic work anytime I hear my baby crying outside whilst in class. Although I pretend most at times to be concentrating in class, its simply not possible but I just have to fake the concentration just to please my lecturer—(Diploma; Student- mother)*

***Mechanisms for coping with childcare and academic work***

Another respondent indicated that

*Due to differences in timetable, my friends sometimes care for my baby when I go for lectures……..* *My mother keeps the baby and alerts me through phone calls when my baby is crying or disturbing*.

Another respondent stated that:

..My husband has been really supportive. He normally goes for night duties to ensure that he is always around during the day to support with the child feeding although we had to resort to baby feed because it’s not possible to do exclusive breast feeding on campus. (*Diploma Student -Mother*)

***Overall effect of lack of breastfeeding support on breastfeeding***

*…So where lies the children’s rights policy of Ghana and its implementation when children even in a University environment where these policies are thought can be openly seen to violate the fundamental rights of privacy to at least breastfeeding of these young children*?. (Student mother)

Others were:

…. *The situation would have been better if mothers were heads and directors in the university. They would have understood our plights better.. (Post graduate Diploma Student- mother).*
